# Supplementary material for: Octogenarian patients and laser-assisted lead extraction: Should we put a limit?
Source: PLoS One. 2023 Oct 20;18(10):e0284802. doi: 10.1371/journal.pone.0284802 (PMC10588874; doi:10.1371/journal.pone.0284802)
Supplement: Attachment — Submitted filename: Review 5.0.docx [file pone.0284802.s002.docx]

Dear Redoy Ranjan,

We thank the editors for giving us the opportunity to review our manuscript. As recommended by the editors, we performed a professional English language editing of the paper and we hope that we reach the journals standard. The Introduction section was already rewritten to avoid any overlap with own previous works. If you see any further text reproduction, please indicate it to me. Also, the previous similarities in the introduction section were with another paper talking a completely different issue in the field of CIED. So, the topic is different and because of that I can not provide advancement to the previous work. Regarding the data availability, I uploaded a data table for data availability.

We thank the reviewers for the constructive comments on the manuscript. We will detail in our response below how we plan to address the reviewer comments.

**Reviewer#1**

Comment #1:

We are not told if there were patients who had an indication for lead extraction and were not done for whatever reason. Retrospective studies have inherent bias to what was essentially successful.

Answer to comment #1:

Thank you for your Comment. We as a reference center do not refuse to do operation even in high risk patient as long as we have a correct indication and a patient approval.

Comment #2:

Retrospective studies have inherent bias to what was essentially successful.

Answer to comment #2:

I agree with you.

Comment #3:

The problem with this paper is the small number of patients who are older than 80 years.

Answer to comment #3:

The number of patients over 80 is small But we were glad to have such a number.

Comment #4:

The authors have not done an appropriate literature search before writing their manuscript. Their most recent reference was dated 2019. I strongly advice the authors look at similar publications on the topic with significantly larger numbers. Some of the recent published manuscripts on this subject are below. After reading these the authors need to see if they can add to the literature anything else that has not been observed in these publications.

Answer to comment #4:

We thank you for your concerns. Our comments to the publication are noted below:

*Transvenous lead extraction: The influence of age on patient outcomes in the PROMET study cohort.*

*Akhtar Z, Elbatran AI, Starck CT, Gonzalez E, Al-Razzo O, Mazzone P, Delnoy PP, Breitenstein A, Steffel J, Eulert-Grehn J, Lanmüller P, Melillo F, Marzi A, Leung LWM, Domenichini G, Sohal M, Gallagher MM.*

*Pacing Clin Electrophysiol. 2021 Sep;44(9):1540-1548. doi: 10.1111/pace.14310. Epub 2021 Aug 5.*

*PMID: 34235772*

This also retrospective study showed the 30-day mortality was most significant in the octogenarian and least in the young patients. However, our data showed similar mortality in both groups.

*Transvenous lead extraction in patients with systemic cardiac device-related infection-Procedural outcome and risk prediction: A GALLERY subgroup-analysis.*

*Chung DU, Burger H, Kaiser L, Osswald B, Bärsch V, Nägele H, Knaut M, Reichenspurner H, Gessler N, Willems S, Butter C, Pecha S, Hakmi S; GALLERY investigators.*

This retrospective subgroup study did not analyses octogenarian patients specifically. It analeysd patient with infected CIED as a risk factor.

*Safe and effective transvenous lead extraction for elderly patients utilizing non-laser and laser tools: a single-center experience in Japan.*

*Okada A, Tabata H, Shoda M, Shoin W, Kobayashi H, Okano T, Yoshie K, Kato K, Saigusa T, Ebisawa S, Motoki H, Kuwahara K.*

This retrospective study with a smaller patients’ groups as our showed promising results that support our finding. However, the study involved both laser and mechanical sheaths and as mentioned the groups were smaller.

*Indications for transvenous lead extraction and its procedural and early outcomes in elderly patients: a single-center experience.*

*Ząbek A, Boczar K, Dębski M, Pfitzner R, Ulman M, Holcman K, Kostkiewicz M, Musiał R, Lelakowski J, Małecka B.*

*Pol Arch Intern Med. 2020 Mar 27;130(3):216-224. doi: 10.20452/pamw.15182. Epub 2020 Feb 10.*

*PMID: 32041927*

This retrospective study comparing two groups of young and octogenarian patients showed a with significantly higher non-procedure‑related 30‑day mortality. On the countrary we proved that our patients showed no difference

*Risk Factors and Long-Term Survival of Octogenarians and Nonagenarians Undergoing Transvenous Lead Extraction Procedures.*

*Jacheć W, Polewczyk A, Segreti L, Bongiorni MG, Kutarski A.*

*Gerontology. 2021;67(1):36-48. doi: 10.1159/000511358. Epub 2020 Nov 26.*

*PMID: 33242867*

In this retrospective study, , leads were more often removed using standard extraction techniques: simple traction and mechanical dilatators in octogenarians and laser use in octogenarians was not the focus of the study.

Reviewer#2

Thank you for reviewing our article and for your positive feedback. We will respond to each point respectively below.

Comment #1:

It would be an interesting descriptive study if the authors describe more in detail regarding the reasons of “partial success or failure” in the OG group. This is important because it reflects the bias of treatment leading to the favorable outcome. For example, the procedure was aborted in the OG group after stucking at the SVC level in extracting. This surely would make the procedure safe for the patients and there was no mortality in the OG group. In such a case, that OG patient did not truly undergo extraction to its entirety. It therefore does not support the conclusion the authors made that it is safe for octogenarians to undergo this high-risk procedure. If so, it should be mentioned in the discussion part. For CS lead and dual-coiled ICD, they pose significant risks if the lead has been dwelling in the body for more than 10 years, but it might not have a significant risk or be too difficult to extract if the duration of the implantation was less than that.

Answer to comment #1:

Lead laser extraction is a challenging procedure and the complexity of lead extraction varies for patient to another. We cannot, from statistical view, involve such variations in a study which is supposed to be based on clear facts. The partial success per patient was defined as the inability to remove all leads. In the case of the octogenarian with failure to extract the leads, the laser sheath was advanced over the RV-lead down to the right ventricle. The cause for failure was that we could not free the tip of the lead and so we could not remove the lead. This means, that the laser sheath passed all the susceptible areas in the venous system without causing any complication. Because of that, we thought it is fair enough to include the patient in the study. About the comment on the CS and dual coil lead, we agree we your sightful comment.

Comment #2:

2. Table 1 should provide more detail in comorbidities e.g. prior PCI, CABG (might lower the risk of extraction in some patients due to the lower risk of cardiac tamponade esp if the tear occurred at the SVC level where pericardium still covers).

Answer to comment #2:

Thank you for the comment. Adding new variable to the study as PCI and CABG means we have to go through the entire data again to collect these variables which is very time-consuming and we did not believe it could have an influence on the results. We believe that having a previous PCI does not protect a against laser complications. On the other hand, a previous CABG could reduce the risk of laser. However, a perforation to the right pleura cavity through the subclavian vein or the superior vena cava could fatal.

Comment #3:

In the discussion part, the limitation of the study should be discussed more in detail as this is a singer-centered experience which may not be generalizable to other centers with different setup/experience in extraction procedure.

Answer to comment #3:

Thank you for the advice. We added a new section titled study limitations based on your comment.

Comment #4

The statement: Only one patient suffered from SVC tier [the authors might misspell it; should be “tear”??]. Please describe in detail about this case; OG or YG group, dual-coil ICD lead or other types of the lead, how old the lead was, etc.

Answer to comment #4:

Thank you for the comment. We corrected the spelling mistake. In this patient, two leads were extracted. One lead was a right atrial lead and the other was a right ventricular pacing lead. Both leads were 60 month old. After lead extraction. A new pleural effusion was detected and the patient became unstable. After sternotomy, a superior vena cava tear was found. We added this to the manuscript.

Commnet #5:

One patient died due to pulmonary embolism; this one is in the OG or YG group? Please describe in detail how/when it happened in the postoperative period and what was a likely etiology of this mortality.

Answer to comment #5:

Thank you for the comment. This patient had a vegetation on the tricuspid valve which embolized to the lungs. We added this to the manuscript. The patient became unstable 2 hours after lead extraction and resuscitated in the intensive care and then token to the operation theater where he underwent open thrombectomy followed by ECMO implantation but he eventually died.

Reviewer #3:

Thank you for reviewing our article. Below you will and the answer to your kind comments

Comment #1:

Tables can be arranged in a simpler form. Inclusion of graphs and charts would be more effective in providing a visual comparison between the groups. This is specifically in reference to tables 3 and 4.

Answer to comment #1:

Thank you for the advice. We added charts to tables 3 and 4 as you suggested.

Comment #2

How do you explain the difference in death rates between your study and 0.3% in previous studies?

Answer to comment # 2:

This may be due to the long-standing experience in this field and the standard that we put and the good trained stuff to early detect any complications. Also, this is a small series so that it might represent the whole death rate overall.

Comment #3:

Where does your study account for risk factors of complications mentioned in discussion? Risk factors mentioned included body mass index, female sex. Were they accounted for during the selection process for the study? Were pts with lower mass index, female and having longer ICD implants excluded from selection for a laser extraction?

Answer to comment #3:

In. this study we didn’t make any selection. All the patient who underwent laser lead extraction were included. After that, we analyzed the patients data to find the risk factor. So no one was excluded.

We hope the we answered all the questions properly.

With best regards.

Sameer Al-Maisary
