# Supplementary material for: Octogenarian patients and laser-assisted lead extraction: Should we put a limit?
Source: PLoS One. 2023 Oct 20;18(10):e0284802. doi: 10.1371/journal.pone.0284802 (PMC10588874; doi:10.1371/journal.pone.0284802)
Supplement: Attachment — Submitted filename: Review 2.docx [file pone.0284802.s003.docx]

**Dear Redoy Ranjan,**

We thank the editors for giving us the opportunity to re-review our manuscript. As recommended by the editors, we performed a professional English language editing of the paper and we hope that we reach the journals standard. We thank the reviewers for the constructive comments on the manuscript. We will detail in our response below how we plan to address the reviewer comments.

**Reviewer#4**

**Comment #1:**

The topic of the study is important because, according to the common opinion, TLE in patients > 80 years old may be more dangerous and some patients are disqualified for TLE only because of their age. There are many publications on this topic, many of which use more conventional lead extraction techniques, but with similar results. The study is based on the small number of patients >80y (38 patients), however, a certain advantage of it is a uniform (laser) extraction technique and performing procedures in organizational conditions ensuring maximum safety. In everyday practice, the stepwise approach and cross-over strategy (from simple traction, utility polypropylene sheaths of different sizes, and in the absence of progress of lead dilatation – up to utility mechanical rotational threaded tip sheaths or laser sheaths) are more often used. The authors' results confirm previous reports, enriching our knowledge that also extraction of leads using the laser technique is not more dangerous in octogenarians than in younger patients.

**Answer to comment #1:**

**Thank you for this comment. Adding to the existing knowledge is our goal and we try to optimize our techniques.**

**Comment #2:**

TLE appears to be easier in the elderly. Other authors explain this by a weaker reaction of the endothelium of veins and heart structures to the presence of leads, less intensive development of connective tissue scar around the lead and less intense processes of its mineralization and calcification.

**Answer to comment #2:**

**I agree with you.**

**Comment #3:**

It seems that if the available material is modest, the discussion should be based on publications containing a much larger number of 80- and 90-year-olds.

I suggest that you pay attention to such reports as:

Jacheć W, Polewczyk A, Segreti L, Bongiorni MG, Kutarski A. Risk Factors and Long-Term Survival of Octogenarians and Nonagenarians Undergoing Transvenous Lead Extraction Procedures. Gerontology. 2021;67:36-48. (80-90y -549 patients and over 90 years of age -35 patients).

Giannotti Santoro M, Segreti L, Zucchelli G, Barletta V, Fiorentini F, Di Cori A, De Lucia R, Bongiorni MG. Transvenous lead extraction: Efficacy and safety of the procedure in octogenarian patients. Pacing Clin Electrophysiol. 2020;43:382-387 (202 patients >80)

Kutarski A, Polewczyk A, Boczar K, Ząbek A, Polewczyk M. Safety and effectiveness of transvenous lead extraction in elderly patients. Cardiol J. 2014;21:47-52. (192 patients over 80y)

Burger H, Hakmi S, Petersen J, Yildirim Y, Choi YH, Willems S, Reichenspurner H, Ziegelhoeffer T, Pecha S. Safety and efficacy of transvenous lead extraction in octogenarians using powered extraction sheaths. Pacing Clin Electrophysiol. 2021;44:601-606 (71 patients over 80y)

**Answer to comment #3:**

Thank you for suggestions. We added these publications and discussed it in the discussion as you proposed.

**Reviewer#5:**

**Comment:**

Lead extraction procedures have increased in recent years due to the augmented number of cardiac implantable electronic devices, a term which includes implantable cardioverter-defibrillators (ICDs)

and permanent pacemakers (PMs). The main reasons for lead extraction are infection and malfunction of the device and/or catheters either after a first implant or a substitution/upgrade procedure.

The paper is a interesting paper. Is ok the remember that the indication for the lead extraction in not relatel to the age of the patients. Is possible to accept with the corrections.

**Answer to comment:**

Thank you for reviewing our article. We did our best to correct the pitfalls to reach a high standard publication.

**Reviewer #6:**

**Comment #1:**

In this manuscript authors report a retrospective comparison of two population (one of octogenarian patients, one of patients < 80 years old) undergoing TLE for various indications. Study aims are potentially interesting as the target demographics for this kind of procedure has progressively aged in recent decades. However, this manuscript holds several significant flaws in study ideation, conduction and reporting. English language needs major revision before consideration.

**Answer to comment #1:**

We thank you for your concerns. We did a detail revision of the paper and corrected the grammatic mistakes as you advised.

**Comment #2:**

Study population, especially for the target demographics (patients < 80 years old) is small, significantly reducing result generalizability.

**Answer to comment #2:**

We agree we you that the study population is small, but this is because the number of octogenarians undergoing laser extraction is not huge and the participation of every experience will enrich our knowledge to better treat these patients.

**Comment #3:**

Evidence regarding this topic is broadly available in papers with higher standards and larger populations:

- https://www.sciencedirect.com/science/article/pii/S0914508719302953

- https://pubmed.ncbi.nlm.nih.gov/23799557/

- https://pubmed.ncbi.nlm.nih.gov/33394103/

- https://www.ahajournals.org/doi/full/10.1161/CIRCEP.111.964270

- <https://www.karger.com/Article/Abstract/511358>

**Answer to comment #3:**

Thank you for mentioning these papers. We will discuss each one below:

- https://www.sciencedirect.com/science/article/pii/S0914508719302953

Atsuhiko et al discussed the safety of laser lead extraction in Japanese population with a low BMI and a smaller collective in comparison to our study which has more collective and normal to slightly elevated BMI.

- https://pubmed.ncbi.nlm.nih.gov/23799557/

Kutarski et al paper was included in our discussion in its reviewed version. It has showed good result after mechanical extraction. No laser was used.

- https://pubmed.ncbi.nlm.nih.gov/33394103/

This retrospective study with a smaller patients’ groups as our showed promising results that support our finding. However, the study involved both laser and mechanical sheaths and as mentioned the groups were smaller.

- https://www.ahajournals.org/doi/full/10.1161/CIRCEP.111.964270

This study from 2011 also investigated the safety of TLE in octogenarian. The authors reported 100% success in all patient but did not mention how many leads were left. They mentioned the removed leads. I think at that time, Laser extraction was a new tool and over the years, our experience has advanced and our approach also. So this paper is relatively good for that time but did not give us the perspectives that we need now.

- https://www.karger.com/Article/Abstract/511358

Jacheć et al paper is included n the revised version of the paper. It investigated mechanical lead extraction.

Comment #4:

Methods: “Consecutive 270 patients” should say “270 consecutive patients”

Answer to comment #4:

Thank you for your correction. We change the sentence as you adviced.

Comment #5:

Results: apart from mortality, briefly, other complications should be cited.

Answer to comment #5:

Thank you for the comment. We added the other complications to the abstract.

Comment #6:

Conclusion: “In octogenarian” should say “In octogenerians”

Answer to comment #6:

Thank you for the comment. We checked the spelling and it was correct so we did not change it.

Comment #7:

The of redness” should be rephrased

Answer to comment #7:

Thank you for the correction. We rephrased the sentence.

Comment #8:

Statistical analysis should include all statistical tests used

Answer to comment #8:

Thank you for the comment. We included the used statistical tests to the methods.

Comment #9:

Please define “renal insufficiency”

Answer to comment #9:

Thank you for the comment. We added a definition to the results section.

Comment #10:

“Lead demographics” table should be renamed to “Lead characteristics”, are numbers relative to dwelling time in months? There is no description or legend.

Answer to comment #10:

Thank you for the comment. We changed lead demographics to lead characteristics. The dwelling time is in months. we only added (in) to increase the clarity.

Comment #11:

Figure 1-2-3: please add numerical values, graphs are not useful as presented.

Answer to comment #10:

Thank you for the comment. We added numerical values to the graphs.

**We hope the we answered all the questions properly.**

**With best regards.**

**Sameer Al-Maisary**
